# Scratching beyond the surface: examining macroecological patterns in avian eggshell texture

**DOI:** 10.1098/rsif.2024.0527

**Published:** 2025-11-26

**Authors:** Marie R. G. Attard, James Bowen, Steven J. Portugal

**Affiliations:** ^1^ Department of Biological Sciences, School of Life and Environmental Sciences, Royal Holloway University of London, Egham, Surrey TW20 0EX, UK; ^2^ School of Engineering & Innovation, Open University, Milton Keynes MK7 6AA, UK; ^3^ British Antarctic Survey, Natural Environment Research Council, Cambridge, Cambridgeshire CB3 0ET, UK; ^4^ Department of Biological Sciences, The University of Oxford, Oxford, Oxfordshire OX1 3SZ, UK; ^5^ The Natural History Museum, Tring, Herts HP23 6AP, UK

**Keywords:** avian, kurtosis, roughness, skewness, surface structure

## Abstract

The surface texture of bird eggshells differs remarkably between species and is thought to play a substantial role in providing physical and microbial protection for the developing embryo. We used high-resolution optical profilometry to establish the key evolutionary drivers of surface textural diversity in eggshells from 453 bird species across 98 families. Within a phylogenetically informed framework, we aimed to determine which life-history traits and nesting environments probably determine eggshell surface texture. We measured surface roughness (*S_a_*, nm), surface skewness (*S_sk_*) and surface kurtosis (*S_ku_*), which describe different aspects of the properties of eggshell surface texture. *S_a_* represents the average height variations on the surface, providing a measure of smoothness or roughness. In contrast, *S_sk_* reveals the distribution of surface features, where positive values signify a predominance of peaks, while negative values indicate a greater presence of valleys. Lastly, *S_ku_* assesses the geometry of these features, with values exceeding 3 suggesting the presence of sharp peaks or deep troughs, and values below 3 indicating a flatter, more uniform surface. Overall, eggshell surfaces were smoother among species that lay immaculate eggs, meaning those without any pattern, in contrast to maculate eggs. Eggshells from semi-enclosed nests had smoother surfaces than those laid in exposed (cup, bowl, platform, no nest) nests. We found that 90.1% of the species had eggshell surfaces mainly composed of valleys rather than peaks, based on their *S_sk_*. By exploring the properties and performance of porous surfaces in nature, we may inspire future biomimicry designs that take advantage of these discoveries.

## 1. Introduction

The eggshell of birds is a biomineralized composite ceramic made up of calcium carbonate within an organic matrix [1,2]. The covering deposited on the outermost surface of the eggshell—the cuticle—is present in all but a few bird lineages (parrots, pigeons and petrels), and probably performs multiple simultaneous functions, none of which are mutually exclusive [3]. The cuticle can act like a seal, moderating gas exchange and the loss of metabolic water from inside the egg to the external environment [3,4]. The cuticle also influences how readily external microbes can penetrate the shell [3]. In some species, the cuticle is permeable to water vapour and gases through cracks forming over the pore opening (e.g. European herring gulls, *Larus argentatus*) [2,5]. In other species, the cuticle can limit eggshell conductance by blocking pores, either through forming a uniform cap over pores (e.g. domestic fowl, *Gallus domesticus* [1,2,6]) or plugging pore openings (e.g. greater rheas, *Rhea americana* [1,2,7]). However, it is important to note that the effect of cuticle removal on eggshell conductance is not universally significant across all species, as demonstrated in experiments using several domestic bird species [8]. Several studies suggest that cuticle characteristics are adapted to specific habitats and incubation environments, and may influence the hatchability and incubation period of the egg [3–5,9,10]. Such parameters can influence the roughness of the eggshell surface [11]. Eggshell roughness refers to the three-dimensional features of the eggshell surface [2,3,12]. Eggshell roughness is typically greater in the foreground pigmentation than the background in species which lay maculated eggs, and the background region of maculated eggs is more similar in surface topography to the surface of immaculate eggs from different species [13,14]. While prior work has established the differences in roughness between foreground and background pigmentation in eggs [14], overall eggshell roughness across multiple species has yet to be studied.

External influences such as wider habitat and nest environment can dictate avian egg survival [15,16]. The eggshell itself is home to a diverse array of microbiota that differ in their ability to penetrate the shell and subsequently infect the developing embryo, potentially causing death [17]. Opportunistic saprophytes such as gram-positive cocci, gram-negative enterics, gram-negative fermenters and certain fungi, are the main causes of egg infections [6,18]. These include certain pseudomonads and fungi that can digest the cuticle layer, destroying the egg’s water-resistant properties and increasing the number of unplugged pores available for trans-shell transmission [6,18]. Birds nesting in wetter and warmer habitats are generally at a higher risk of microbial infection, resulting in a prevalence of anti-microbial structures on the shell surface in these species [15]. Contact incubation can help minimize the build-up of harmful microorganisms by reducing moisture on the egg or by the antimicrobial properties of certain waxes and fatty acids found on the feathers of the incubating parents, in their preen gland secretions and within the epidermal layer on the brood patch [19–22]. Species nesting in burrows and arboreal cups—enclosed environments—are exposed to greater humidity levels, and these species exhibit higher eggshell conductance to counteract the greater moisture level, in comparison with those species nesting on the ground or in shallow arboreal nests [13,23]. Given these clear distinctions in conductance and microbial risk between different habitats and nest types, we predict that specific eggshell surface structures will be shared among species with similar nest environments.

While there are macroevolutionary studies investigating the influence of climate and life history of birds on eggshell pigmentation [14], none have quantified the variation in eggshell surface texture across multiple species, or sought to elucidate the primary determinants driving this variation. Studies on non-avian reptiles, such as that of D’Alba *et al.* [24], have provided important insights into the evolution of eggshell structure in relation to nesting ecology. Their work quantified egg shape, shell thickness, porosity and mineralization across a broad range of reptilian species, offering valuable context for understanding eggshell evolution across different vertebrate groups. However, D’Alba *et al*.’s study did not examine eggshell surface texture, leaving a gap in our knowledge, particularly in avian species. Generally, surface structures can alter multiple paramaters, with smoother eggshell surfaces being associated with glossier eggs [25] and reduced microbial adhesion being documented on the rougher surfaces of structures such as the leaves of the lotus plant (*Nelumbo nucifera*) [26]. A comparative study found the surface of maculated eggs consisted of a rougher foreground pigment compared with the background pigment across 71% of 204 bird species (54 families) studied. While this study [14] investigated the within-egg difference between foreground and background eggshell, it did not compare overall surface roughness between species. Large-scale macroecological studies of overall eggshell texture will, therefore, further improve our understanding of the importance of the nest structure, incubation behaviours and climatic factors in shaping the evolution of avian eggshells.

We quantify the external morphological attributes of a wide diversity of avian eggshells to determine which life-history traits are associated with variation in eggshell surface texture between bird species. To achieve this, we compiled high-resolution three-dimensional scans of avian eggshells to characterize their surface structure. We examined ecological theories concerning how changes in texture of the eggs across various lineages fluctuate with differences in nesting environments and pertinent life-history traits. Drawing on a comprehensive review of existing literature, we investigated several physical and life-history factors that could account for the variation in roughness among species (as outlined in table 1).

**Table 1.** Putative predictions, rationale and definitions for possible explanations for variation in eggshell topography in birds. Source lists references for definitions, and primary databases used to compile bird life-history traits. Hypotheses for variation in eggshell topography are differentiated as either a proximate or ultimate cause. Ultimate explanations address evolutionary function (i.e. why eggshell topography exists) and proximate explanations address the way in which the functionality is achieve (i.e. how inter-specific differences in surface topography are achieved by the eggshell). These two types of explanations complement each other and are not mutually exclusive. Hypotheses are numbered 1 to 15.

ID	predictor	cause	hypothesis	definition	source
1	body mass	proximate	surface roughness is reported to increase with organism size. As adult body mass is correlated to egg mass, eggshells of heavier birds will have a rougher surface.	mean body mass (g) of adult birds.	Data from Dunning [27], with updates from Wilman *et al*. [28] and Pigot *et al*. [29]. Database compiled by Sheard *et al*. [30].
2	clutch size	ultimate	evaporation from multiple eggs will create a nest atmosphere of greater humidity, so water contact angle will be higher for species with larger clutches. Smoother eggshells among species with larger clutches would help reduce microbial infection.	number of eggs per brood, measured as geometric mean of the typical minimum and maximum clutch size.	Databases from Jetz *et al.* [31], Lislevand *et al*. [32] and Myhrvold *et al*. [33]. Gaps filled in using HBW Alive [34] and other sources.
3	incubation period	ultimate	species with longer incubation periods will be rougher than expected for their body mass to reduce water loss from the egg.	average number of days an egg is incubated.	Data from Myhrvold [33]. Gaps filled in using HBW Alive [34] and other sources.
4	maculation		species with immaculate eggshells will have smoother surfaces than species with maculated eggs, based on differences in surface topography of foreground and background pigments.	**(1) immaculate:** no foreground pigment **(2) maculate:** foreground and background pigment	Data from Attard *et al*. [14] and references therein.
5	diet	proximate	the composition of the eggshell is influenced by diet. Plants and insects have low calcium content, so species that rely on these foods are expected to have thinner eggshells compared with species that feed on vertebrates. As microbes can more easily enter the egg of thinner eggshells, species that consume plants or insects will develop smoother eggshell surfaces to minimize microbial adhesion.	**(1) plant:** diet primarily consists of fruit, buds, seeds or plants **(2) insectivore**: diet primarily consists of insects **(3) omnivore:** diet is omnivorous **(4) carnivore/scavenger**: diet is carnivorous or a scavenger	Category based on Wilman *et al*. [28], updated from HBW Alive [34] and other sources. Database from Sheard *et al*. [30].
6	mode of development	ultimate	longer incubation duration will promote the accumulation of microbes on the eggshell surface. Precocial species require more incubation time than altricial species, thus are expected to possess smoother eggshell surfaces.	**(1) altricial:** newly born young are relatively immobile, naked, and usually require care and feeding by the parents. **(2) precocial:** newly born young are relatively mobile, covered in feathers, and independent.	Category based on Augustine *et al.* [35], Stark [36] and Stark & Ricklefs [37]. Data from HBW Alive [34] and other sources.
7	nest type	ultimate	nests in cavities or burrows have a higher relative humidity than open-top nests and are more insulated. As the level of bacterial penetration through the shell increases with higher temperature and relative humidity, the shell surfaces of eggs laid in enclosed nests will be smoother than eggs laid in semi-enclosed and exposed nests.	**(1) exposed:** nest is open above and has no side walls (no nest, scrape, saucer, platform, heap). **(2) semi-enclosed:** nest is partially open and has side walls (cup, bowl, pendant, sphere, dome, pouch). **(3) enclosed:** nest is entirely enclosed (cavity, burrow, crevice).	Category from this paper. Data from HBW Alive [34] and other sources.
8	nest location	ultimate	elevated nests have lower risk of flooding, water accumulation or exposure to dirt and animal faeces, therefore their eggshells will be rougher compared with burrows and ground-nesting species, due to reduced risk of infections.	**(1) ground:** nest location in or on the ground. **(2) water:** floating on water. **(3) elevated:** nest located in tree, bush, shrub, wall, cave roof, cliff or attached to reed.	Category based on Portugal *et al.* [23]. Data from HBW Alive [34] and other sources.
9	habitat	ultimate	eggs of species breeding in open habitats are more vulnerable to heat loss due to exposure to wind, therefore their eggshells are expected to have smoother surfaces to reduce heat loss compared to eggs of species breeding in semi-open and dense habitats.	**(1) open:** species primarily occurs in desert, grassland, open water, open moorland, low shrubs, rocky habitats, seashores and cities. **(2) semi-open:** species primarily occurs in open shrubland and bushland, scattered bushes, parkland, forest edge. **(3) dense:** species primarily occurs in forest with a closed canopy, or in the lower vegetation strata of dense thickets, shrubland, mangroves or marshland.	Habitat scores from Tobias *et al.* [38]. Database compiled by Sheard *et al.* [30].
10	nest lining	ultimate	incorporation of nest lining will trap moisture, resulting in smoother eggshell surfaces to repel water.	**(1) lined:** nest lining is always or sometimes present. **(2) not lined:** nest lining is absent.	Category from this paper. Data from HBW Alive [39] and other sources.
11	incubating parent	ultimate	eggs are more prone to microbial penetration when the parent leaves the nest uncovered. This is more likely to occur if incubation is not shared between parents, hence these eggs are more likely to have smoother eggshells.	**(1) not shared:** contact incubation of eggs by single adult. **(2) shared:** contact incubation of eggs by two adults.	Category from Portugal *et al* [23]. Data from HBW Alive [34] and other sources.
12	parental contact	ultimate	the wet incubating parent returning to the nest will increase the nest’s humidity, thus eggshells of these species are expected to have smoother eggshells.	**(1) wet plumage:** adults return habitually to the nest with wet plumage. this included species that feed on freshwater or marine prey, or use nests built on water. **(2) dry plumage**: adults did not return habitually to the nest with wet plumage.	Category from Portugal *et al.* [23]. Data from HBW Alive [34] and other sources.
13	parental care	ultimate	the eggshells of species that provide biparental care are expected to have rougher texture, as nest humidity and temperature can be better maintained when both parents assist.	**(1) uniparental:** the brood is provisioned and/or defended by one adult **(2) biparental:** the brood is provisioned and/or defended by at least two adults	Category from Portugal *et al.* [23]. Data from HBW Alive [34] and other sources.
14	annual temperature	ultimate	as the level of bacterial penetration through the shell increases with higher temperature, eggshells of eggs incubated in warmer climates will be smoother to avoid microbial colonization.	average annual mean temperature (BIO1) of breeding/resident range.	From Sheard *et al.* [30], based on WorldClim v1 data [40].
15	annual precipitation	ultimate	Eggshells incubated in environments with higher annual precipitation will be smoother to combat temporary periods of excessive rain.	average annual mean precipitation (BIO12) of breeding/resident range.	From Sheard *et al.* [41], based on WorldClim v1 data [40].

## 2. Material and methods

### 2.1. Overview of samples, sources and preparation

**Figure 1.**
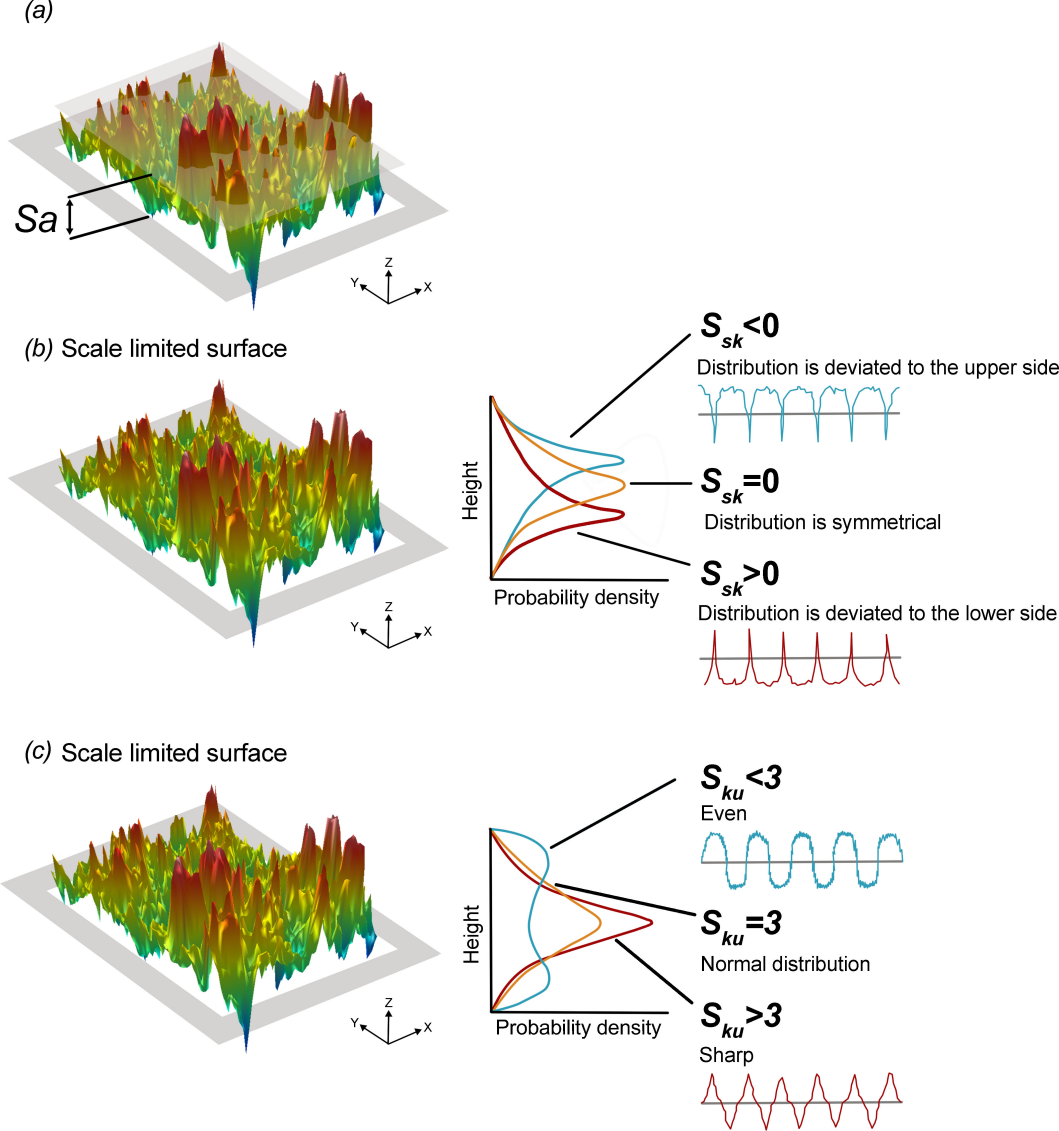
Diagrammatic representation of *S*_*a*_, *S*_*sk*_ and *S*_*ku*_ values on a three-dimensional surface height distribution. (*a*) *S*_*a*_ represents the mean of the average height difference within the evaluation area. *S*_*a*_ is the mean difference in height from the mean plane and is not significantly influenced by scratches, contamination and measurement noise. (*b*) The *S*_*sk*_ parameter represents the degree of symmetry of the surface heights about the mean plan and its sign indicates whether peaks (i.e. *S*_*sk*_ > 0) or valley structures (*S*_*sk*_ < 0) dominate the surface. (*c*) *S*_*ku*_ describes the tip geometry of the peaks and troughs; *S*_*ku*_ > 3 indicates the presence of inordinately high, sharp peaks or deep troughs while *S*_*ku*_ < 3 would indicate that the tip geometry of the peaks and valleys are rounded. Adapted from Herring *et al*. [34].

Three measures of eggshell surface texture were obtained for 453 avian species; surface roughness (*S_a_*, nm), surface skewness (*S_sk_*) and surface kurtosis (*S_ku_*) (figure 1). S*_a_* represents the mean difference in height from the mean plane [40,42,43], providing stable results as it is not significantly influenced by scratches, contamination and measurement noise [40]. *S_sk_* quantifies the degree of symmetry of surface heights relative to the mean plane, with a positive *S_sk_* (> 0) indicating a dominance of peaks and a negative *S_sk_* (< 0) indicating a dominance of valleys. *S_ku_* describes the sharpness of the peak tips and the depth of the troughs; *S_ku_* > 3 indicates the presence of inordinately high peaks or deep troughs, while *S_ku_* < 3 indicates their absence [43].

All eggshells (total *n* = 1404; see electronic supplementary material for the full list of specimens and sources) were obtained from the Western Foundation of Vertebrate Zoology (WFVZ) in Camarillo, USA, and the Class II destructive collection at the Natural History Museum (NHM) in Tring, UK. The NHM Class II collections contains both intact and broken eggshells, with limited metadata available. By contrast, the WFVZ collection provides publicly accessible information on each clutch (https://collections.wfvz.org/). At WFVZ, we sampled from a designated section of clutches intended for destructive sampling, which contained cracked or broken eggshells, yet retained high-quality data. Nearly all available species from this section were included in our study, ensuring a broad taxonomic range. However, due to restrictions imposed by the COVID-19 lockdown, we were only able to sample a subset of species from the NHM collection prior to enforcement of these restrictions, focusing mainly on passerines.

Our sampling aimed for up to 10 eggs per species when available, selecting a single egg at random from each clutch if more than one clutch was available. Given that there are over 10 000 known species of birds [44], the 453 species included in our study represent a relatively small proportion of avian diversity. However, we did not bias our selection toward specific families or orders; instead, species availability within these extensive collections determined our sampling. While this may limit comprehensive phylogenetic representation, the dataset spans a wide range of taxonomic groups to provide valuable insights into the patterns of eggshell surface texture across avian species.

Our study follows the same protocols as Attard *et al.* [14]; all procedures for sampling, preparing and measuring surface texture of eggshell fragments are described in this paper. In summary, a micro-tool rotary saw with diamond-coated thin cutting wheel (Dremel 4000, Bosch Leinfelden, Germany) was used to extract an approximately 1.5 × 1.5 cm fragment from the equatorial region of the eggshell. All fragments, except those with a vaterite coating [45], were cleaned with distilled water on a cotton bud, then left to air dry.

### 2.2. Profilometry measures of surface texture

The surface topography of eggshell surfaces was obtained using a three-dimensional non-contacting optical profilometer (DCM 3D, Leica Microsystems, Germany) connected to a white light interferometric microscope. For each eggshell fragment, a section along the surface was scanned at three non-overlapping locations at a focal depth of 100 μm (100 focal planes at 1 μm resolution) using the 20× objective magnification to give a measurement area of 636.61 × 477.25 μm^2^ (pixel resolution = 768 × 576). *S_a_*, *S_sk_* and *S_ku_* are based on surface height distribution [46] and are scale-dependent. The same scanning parameters and magnification were applied to all eggshells scanned; therefore, direct comparisons of surface texture between specimens requires that the measurement scale and the sampling interval remain the same.

The foreground and background pigment of maculated eggshells were scanned separately at three randomly selected non-overlapping locations. In contrast, eggshells from immaculate eggs and densely speckled eggs—which were too difficult to divide into foreground and background pigments—were scanned at three randomly selected, non-overlapping locations. Each scan (total 7013 scans) was processed using the scanning probe image processor (SPIP) software (version 4.4.3.0, Image Metrology, Hørsholm, Denmark) to quantify *S_a_*, *S_sk_* and *S_ku_*. We used the plane correction tool to automatically correct plane distortions in the data by using polynomial functions to fit the surface topography. In this case, a second-order polynomial was used as the slope on the data was approximately spherical, then the lowest *z*-value was adjusted to 0 nm. These corrections help eliminate artefacts caused by sample tilt or uneven surfaces, ensuring that the quantitative measurements of surface features are accurate and reliable.

When using an interferometer to scan curved eggshells, we occasionally encountered missing pixel height data due to several factors. The curvature of the eggshell can create varying angles of incidence for the light used in interferometry; if the angle becomes too steep, it may result in low reflectivity and consequently missing data. Additionally, this curvature can cause sections of the surface to fall out of the focal plane or misalign with the beam path, leading to gaps in the pixel data. Our scans were taken under a narrow focal plane, so the centre of the field of view usually captured the topography well and were suitable for inclusion in the analysis, even where pixel information was absent around the scan edges. Since interferometry relies on coherent light, any variations in surface texture can disrupt interference patterns. Rough or imperfect surfaces may generate noisy data or result in the loss of coherence, contributing to missing pixels. To address these issues, we meticulously inspected each eggshell surface scan with less than 40% pixel coverage for irregularities in SPIP. This inspection revealed that areas at the corners or sides of the rectangular scanning region often lacked pixel data, as these sections typically fell outside the focal plane. If sufficient data remained within the focal plane, those scans were cropped to remove low-quality regions for analysis. Conversely, scans exhibiting extensive pixelation were deemed indicative of surface imperfections, such as dirt or other obstructions, leading to noisy data, and were subsequently excluded from our analysis.

All subsequent analysis was performed in the program R v. 4.3.2 (R Software, Vienna, Austria, http://www.R-project.org). Surface texture profiles from brood parasites were excluded from this study as they have specific eggshell adaptations to suit their unique breeding strategy (but see [14,47] to access brood parasite data). We tested the repeatability of each eggshell texture measurement by conducting a repeatability test for multiple measures at different locations on the same fragment (rpt, R package rptR; [48]) The repeatability tests were performed on linear models, in which we set specimen as the random factor with *S_a_*, *S_sk_* and *S_ku_* as the dependant variable. Repeatability of scan measurements was analysed separately for immaculate and maculated eggs, with the latter assessed individually for foreground and background pigments. Scans of maculated eggs were cropped to isolate either the foreground or background pigment. These repeatability tests evaluated variability in surface texture across three or more scans at different locations on the same egg for the maculation or pigment type being analysed.

Surface texture values from multiple locations on the same egg were averaged to obtain a specimen mean value for both immaculate and maculated eggs (*n* = 1404 specimens). Cook’s distance was applied to specimen *S_a_*, *S_sk_* and *S_ku_* values [49] to identify outliers and/or influential values. For species represented by a single egg, any influential specimen was excluded from the analysis. For species represented by multiple eggs, if all specimens were identified as influential, all were retained for analysis. However, if only a portion of a species’ eggs were identified as influential, those influential specimens were excluded from further analysis.

After removing low-quality scans and influential values, surface texture measurements from 1725 specimens across 460 species were retained. *S_a_*, *S_sk_* and *S_ku_* values were first averaged per specimen across retained scans and then averaged at the species level from specimen-specific values for phylogenetic comparative analyses. As the distributions of *S_a_* and *S_ku_* values across species were skewed, we log_10_ transformed these response variables to achieve a normal distribution for statistical analysis. *S_sk_* values were not transformed as they had a normal distribution across species.

### 2.3. Association between surface texture, body mass and wettability

We collected data on 20 key life-history traits (table 1) that have been previously identified in the literature as potentially influencing the surface roughness of avian eggshells. Of these, 15 predictors were included in the analysis due to multi-collinearity (see electronic supplementary material, figures S1 and S3). Hypotheses and definitions of the final predictors are listed in table 1 (but see electronic supplementary material, table S4, for hypotheses for predictors excluded from analysis), and sources used to extract this data are listed in electronic supplementary material and Figshare repository (https://figshare.com/s/1dbaa5ae26554677473d). Species sample sizes for each categorical predictor are detailed in electronic supplementary material, table S5.

Phylogenetic signal in the eggshell surface properties was measured using Pagel’s lambda (*λ*) [50] using the *phylosig* function in the package ‘phytools’ [51]. Pagel’s *λ* (range 0–1) determines to what extent related species were more likely to share similar surface texture values than species drawn from a tree. Pagel’s *λ* values closer to 0 indicate no phylogenetic signal in the data while values approaching 1 are more consistent with a Brownian motion model of trait evolution in which the phylogeny accurately reflects the covariances between species for a given trait [52]. The *phylosig* function was used to test the hypothesis that Pagel’s λ is different from 0. The difference in log-likelihood ratio of the lambda model (*phylosig* function) and Brownian motion model (*brownie.lite* function) was compared with a chi-squared (*χ*^2^) distribution with one degree of freedom to test the hypothesis that Pagel’s λ is different from 1.

As body mass affects all aspects of animal biology and ecology, we ran a phylogenetic generalized least squares (PGLS) model of adult body mass as a predictor of *S_a_*, *S_sk_* and *S_ku_*. Adult body mass was taken from average body mass per species found in the literature. If these relationships were significant, the residual values would be used as the response variable in a second series of PGLS models to remove body mass as a predictor. This second series of models would allow us to determine if one or more life-history traits result in higher or lower *S_a_*, *S_sk_* and *S_ku_* values than is expected for a given body mass of the adult bird. Data from the present study were compared with species-level wettability measurements from Attard *et al.* [53] using a PGLS to determine the relationship between roughness parameters and wettability.

We evaluated if life history explained overall variance in each eggshell surface variable using generalized linear mixed models with Markov chain Monte Carlo (MCMCglmm) estimation methods, implemented in the ‘MCMCglmm’ package [54,55] with an adapted R script by Attard *et al.* [53]. This Bayesian mixed effects approach allowed us to incorporate within-species variation in θc and Δθc [54] by fitting individual-level data while also controlling for non-independences in species traits due to shared evolutionary ancestry [56], enabling the identification of phylogeny-adjusted patterns in species traits [57]. We tested for collinearity among pertinent life-history traits and only selected uncorrelated variables (with paired-correlation less than 0.75) and variation inflation factor (VIF) under 10 as predictor variables in the MCMCglmm (see Attard *et al.* [14,53] for details). *S_a_*, *S_sk_* and *S_ku_* were modelled separately. In the main model, each individual-level surface texture value was set as a dependent variable and the life-history traits were included as fixed effects.

In our MCMCglmm models, phylogeny was included as a random effect, together with species level, to control for phylogenetic non-independence and non-independence due to factors unrelated to phylogeny [56]. The phylogenetic tree was extracted from the global phylogeny of birds using a web-based tool at http://birdtree.org [58]. BirdTree.org’s tree construction method combines a fossil-calibrated backbone with relaxed-clock molecular trees of avian clades. We downloaded the full trees, which included 9993 species, from which we generated 1000 random trees (all with the Hackett backbone [59]). These trees were then summarized into a single consensus tree, applying 130 000 iterations, 100 thinning intervals, and 30 000 burn-in.

To generate predicted values of eggshell surface traits across all possible combinations of life-history predictors, we computed posterior predictions from the MCMCglmm models. This analysis was performed on a high-performance computing (HPC) cluster (SLURM scheduler; 160 GB memory, two cores). While the original MCMCglmm models used higher iteration numbers and different thinning parameters to ensure robust parameter estimation, reduced settings (80 000 iterations, burn-in of 20 000, and thinning interval of 500) were applied solely during the prediction step to manage computational demands. We created a reduced grid of predictor values in which continuous variables were divided into three equally spaced bins and all combinations of categorical variables were included. Predictions were computed in batches of 500 rows to manage memory usage by multiplying the model matrix for each batch with posterior samples of the fixed effects to obtain posterior means and 95% highest posterior density intervals (HPDIs). The results from all batches were combined into a single file (1 048 575 rows representing all possible predictor combinations) for each eggshell surface trait. Predicted effects of significant categorical predictors on eggshell surface texture traits were visualized in R, showing the full posterior density with 50%, 80% and 95% credible intervals and the median, derived from the MCMCglmm predictions.

## 3. Results

### 3.1. Repeatability within eggshell fragments

We found that *S_a_*, *S_ku_* and *S_sk_* were significantly repeatable between locations on the same shell fragment for immaculate eggs (616 specimens and 193 species), and for foreground (505 specimens across 195 species) and background (664 specimens across 212 species) pigment of maculated eggs (electronic supplementary material, table S6). All values from the same fragment were thus averaged to a single specimen value. Mean *S_a_*, *S_ku_* and *S_sk_* were calculated for each species using single specimen values. Outputs for all three eggshell topography indices were independent of each other, with correlation values ranging from −0.12 to 0.14 (electronic supplementary material, table S7).

### 3.2. Surface texture not influenced by body mass

As body mass was not significantly correlated to *S_a_* (PGLS: estimate = −0.012, *R*^2^ < 0.001, *p* = 0.83), *S_ku_* (PGLS: estimate = −0.02, *R*^2^ = 0.01, *p* = 0.23) or *S_sk_* (PGLS: estimate = 0.01, *R*^2^ < 0.001, *p* = 0.81), these response variables did not need to be corrected for body mass to test the influence of other eggshell traits and life-history in the evolution of eggshell texture.

### 3.3. Surface texture associated with spreadability of water droplets on the surface

There were 416 species with wettability [53] and surface texture measures. The initial contact angle of a water droplet on an eggshell decreased with body mass (PGLS: estimate = −5.60, *R^2^* = 0.07, *p* < 0.001), whereas the spreadability of the water droplet across the surface was not associated with body mass (PGLS: estimate = 1.19, *R*^2^ < 0.01, *p* = 0.23). Surface topography did not influence the initial contact angle of sessile water droplets on eggshell surfaces (electronic supplementary material, table S8). However, sessile water droplets spread more on eggshells with rougher surfaces (PGLS: estimate = 0.01, *R^2^* = 0.02, *p* < 0.01).

### 3.4. Phylogenetic signal in average surface topography

Pagel’s *λ* for *S*_*sk*_ was low (*λ* = 0.02) and significantly different from 1 (*p* < 0.001) but not 0 (*p* = 0.43) (table 2). This indicates that variation in *S_sk_* across bird species is largely independent of phylogeny. Pagel’s *λ* for *S_a_* and *S_ku_* were intermediate (*λ* = 0.62 and *λ* = 0.39, respectively) and significantly different from 1 (*p* < 0.001) and 0 (*p* < 0.001).

**Table 2.** Estimates of phylogenetic signal in eggshell surface roughness (S_a_), kurtosis (*S_ku_*) and skewness (*S_sk_*) across all birds (*N* = 460). The *p*-value tests the null hypothesis for both no phylogenetic signal (*λ* = 0) and a Brownian motion model (*λ* = 1) of evolution.

response variable	Pagel’s *λ*	loglikelihood	log-likelihood for *λ* = 0	loglikelihood for *λ* = 1	*p for λ* = 0	*p for λ* = 1
*S_a_*	0.63	−353.79	30.78	−536.96	**<0.001**	**<0.001**
*S_ku_*	0.21	135.06	6.74	−85.37	**0.009**	**<0.001**
*S_sk_*	0.05	−216.42	0.64	−407.63	0.06	**<0.001**

### 3.5. Inter-species variation in surface topography across birds

**Figure 2.**
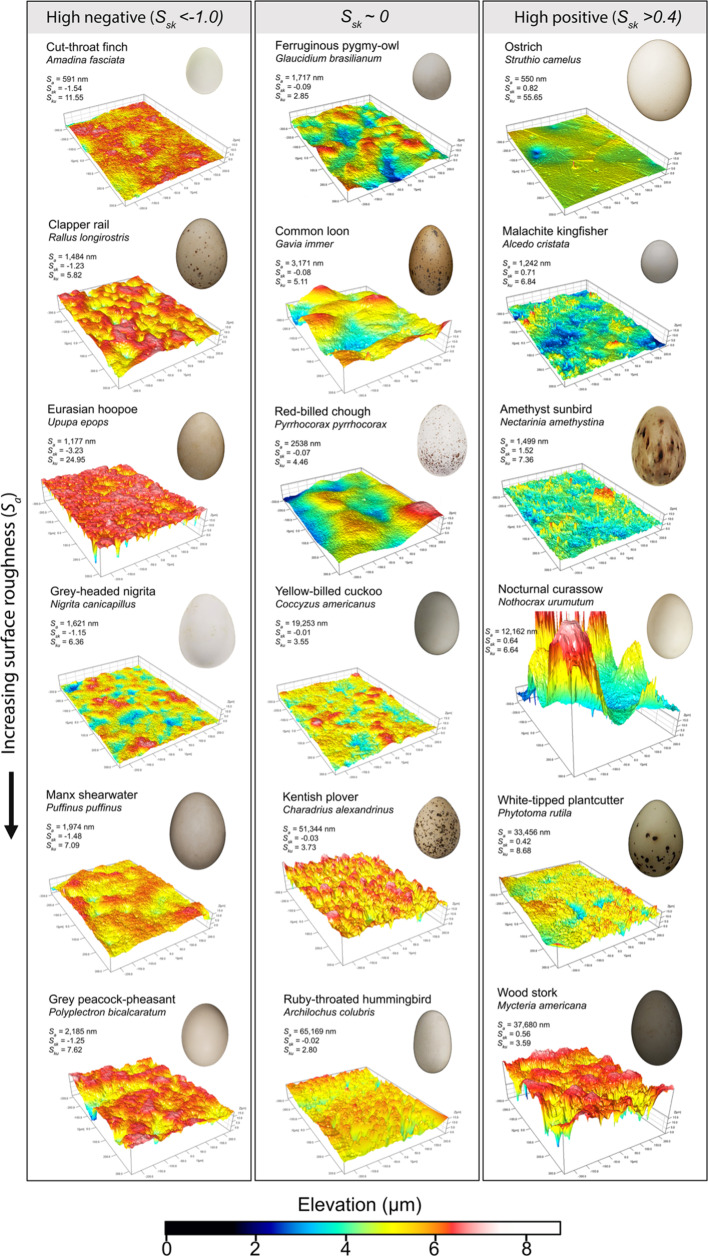
Surface topography of eggshells from a selection of species included in this study. Digital elevation models of the eggshell surface for one specimen per species (1 µm resolution, dimensions 200 × 200 µm). The egg photos are not to scale. More information can be found in the electronic supplementary material.

**Figure 3.**
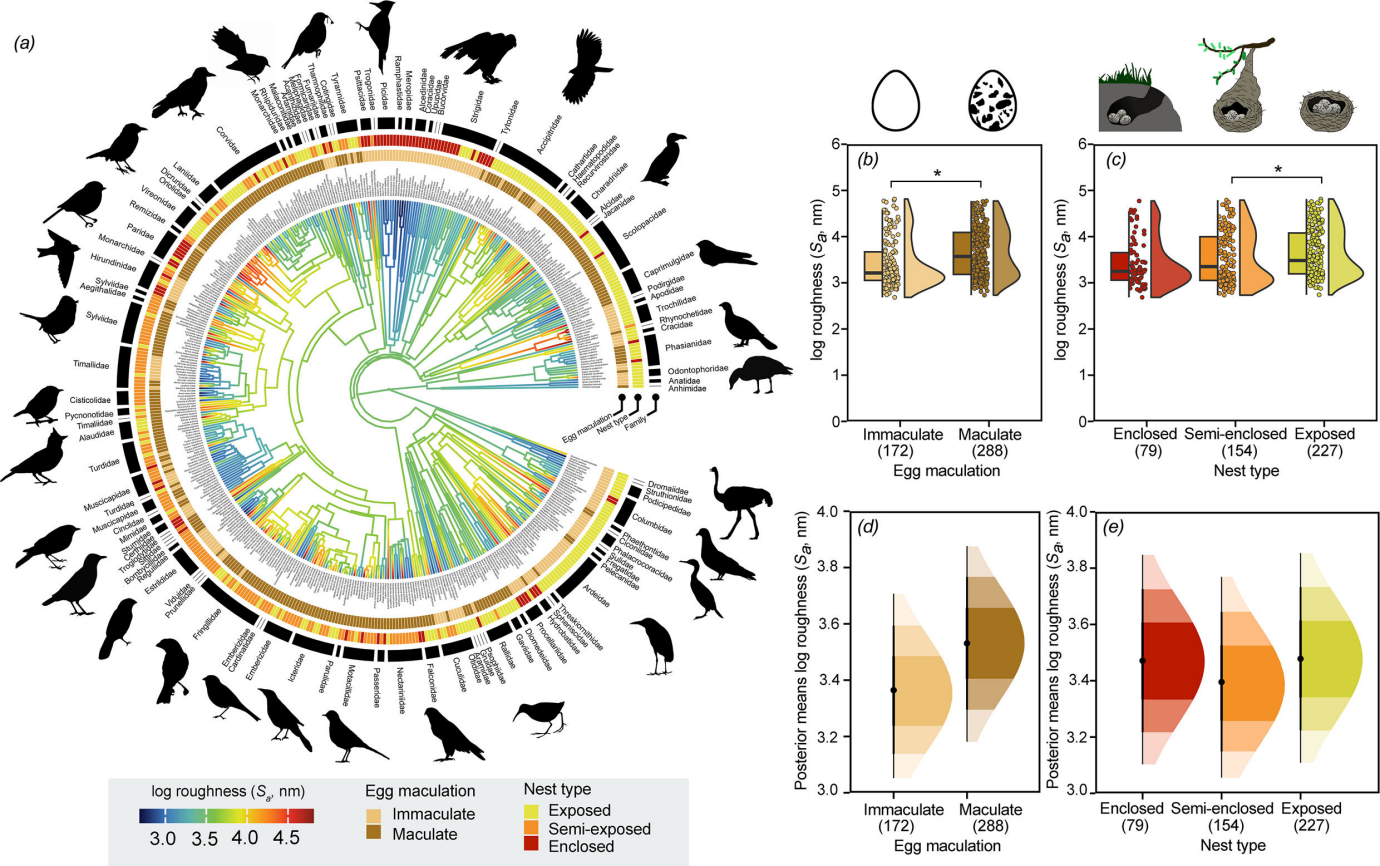
Phylogenetic tree and significant predictors of eggshell surface roughness (*S_a_*) across 460 bird species. *(a)* Branch colours show the diversification in *S_a_* across the phylogeny and branch lengths show ancestral trait estimates. Across all birds, *S_a_* was higher in species that (*b*) laid maculated eggs and (*c*) that use exposed nests compared with species that use semi-enclosed nests. In the hybrid box plots (*c*), species log *S_a_* are shown as filled circles, vertical line indicates the median, box shows the interquartile range (IQR) and the whiskers are 1.5 × IQR, and their distributions are shown as histograms. Significant differences between categorical variables based on MCMCglmm are given by asterisks with *p < 0.05. (*d, e*) Density plots of posterior predictions from significant predictors in the MCMCglmm models with median value and 50%, 80% and 95% credible intervals, illustrating uncertainty around predicted log *S_a_* values for each predictor category. Silhouette illustrations were sourced from PhyloPic (http://phylopic.org), contributed by various authors under public domain licence (see electronic supplementary material).

Two hummingbird species had the highest roughness value across all birds included in the analysis: ruby-throated hummingbirds (*Archilochus colubrisand*, *S_a_* = 65 169 nm, *n* = 1) and hoary pufflegs (*Haplophaedia lugens, S_a_* = 61 622 nm, *n* = 1) (figure 2). Little green bee-eaters (*Merops orientalis: S_a_* = 485 nm, *n* = 1) and common ostriches (*Struthio camelus: S_a_* = 550 nm, *n* = 1) had the smoothest eggshell surfaces. Eggshell maculation and nest type were the main determinants of eggshell surface roughness across birds (figure 3, electronic supplementary material, table S9). Species with maculated eggs had significantly rougher eggshell surfaces than species that lay immaculate eggs (MCMCglmm: *p* < 0.001; electronic supplementary material, table S9) (figure 3*b,d*), and species that use exposed (cups, bowls, platforms, depressions and scrapes) nests had significantly rougher eggshell surfaces than species that use semi-enclosed (dome-shaped) nests (*p* = 0.01; figure 3*c,e*). However, eggshell surface roughness of species that use enclosed (burrow and cavity) nests did not differ significantly from species that lay their eggs in semi-enclosed nests (*p* = 0.17) or exposed nests (*p* = 0.87) (electronic supplementary material, table S9).

**Figure 4.**
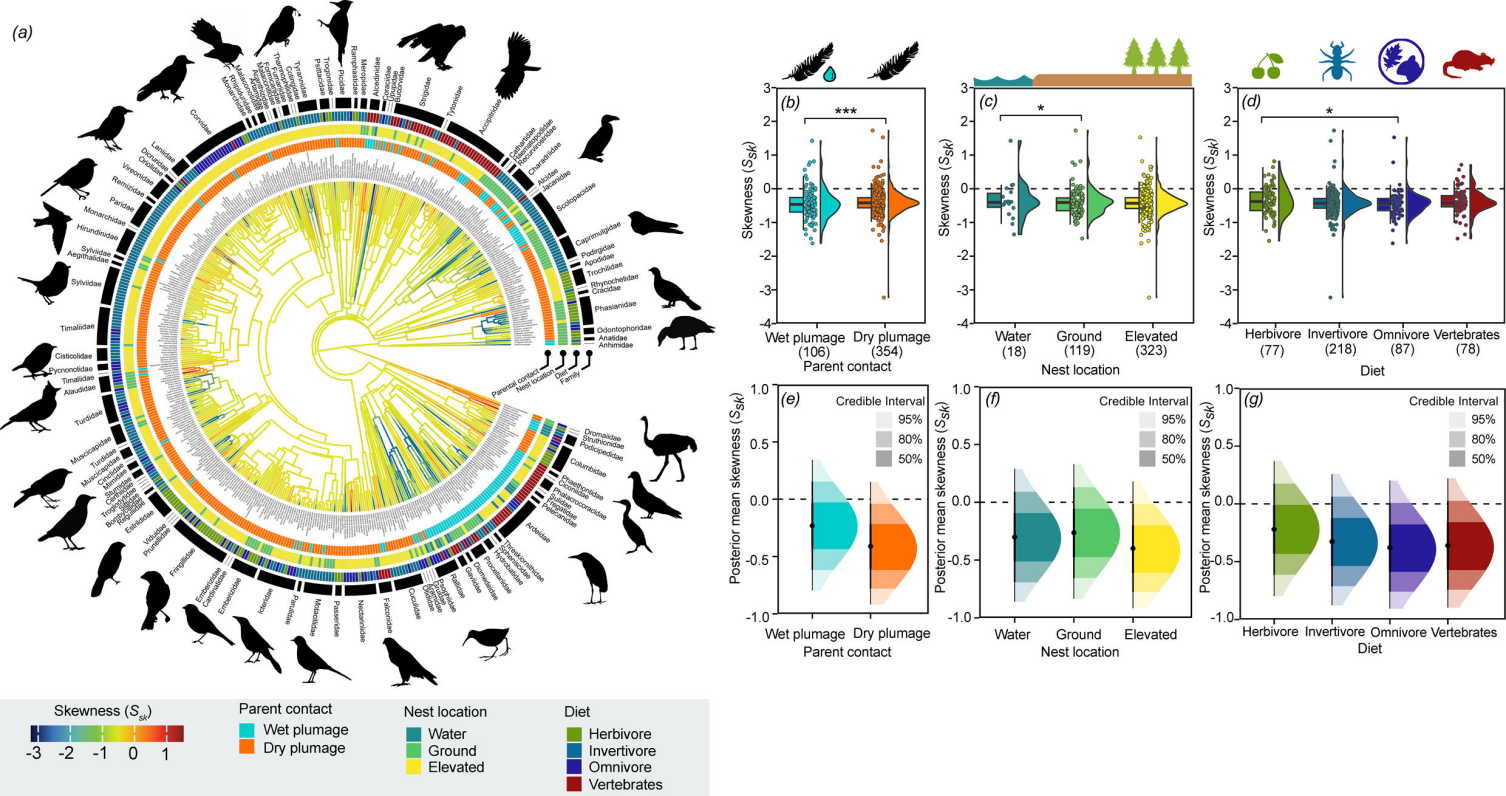
Phylogenetic tree and significant predictors of eggshell surface skewness (*S_sk_*) across 460 bird species. *(a*) Branch colours show the diversification in *S_sk_* across the phylogeny and branch lengths show ancestral trait estimates. *S_sk_* of eggshell surfaces was higher among (*b*) species with wet plumage, (*c*) ground-nesting species and (*d*) herbivores relative to omnivores. In the hybrid box plots (*b–d*), *S_sk_* are shown as filled circles, vertical line indicates the median, box shows the interquartile range (IQR) and the whiskers are 1.5× IQR and their distribution are shown as histograms. Significant differences between categorical variables based on MCMCglmm are given in asterisks with *p < 0.05. (*e–g*) Density plots of posterior predictions from significant predictors in the MCMCglmm models with median value and 50%, 80% and 95% credible intervals, illustrating uncertainty around predicted *S_sk_* values for each predictor category. Silhouette illustrations came from PhyloPic (http://phylopic.org), contributed by various authors under public domain licence (see electronic supplementary material).

We found that 90.9% of the 460 species analysed produce eggshell surfaces with negative *S_sk_*, suggesting these surfaces are mainly composed of valleys rather than peaks. This is unsurprising, given that negative *S_sk_* is characteristic of porous materials [46]. Peaks were particularly dominant on the shell surface (indicated by *S_sk_* > 0) of amethyst sunbirds (*Nectarinia amethystina*; *S_sk_* = 1.52, *n* = 1), black-necked grebes (*Podiceps nigricollis*; *S_sk_* = 1.42 ± 0.35, *n* = 2) and common ostriches (*S_sk_ =* 0.82*, n* = 1) while valleys were most dominant on the shell surface of Eurasian hoopoes (*Upupa epops*, *S_sk_* = −3.23 ± 1.28, *n* = 5). In the MCMCglmm, *S_sk_* was significantly higher for water-nesting species compared with those that nest above ground (*p* = 0.01; figure 4*c*), such as in a tree, bush, wall, cave roof, cliff or attached to reed (electronic supplementary material, table S10). *S_sk_* was also significantly higher for species where parents incubate their eggs with wet plumage (*p* < 0.001; figure 4*b,e*) and in species with plant-based diets compared with those with omnivorous diets (*p* = 0.01; figure 4*d*). These findings were consistent with the posterior mean outputs, revealing similar patterns of association between predictor levels (figure 4*f,g*).

**Figure 5.**
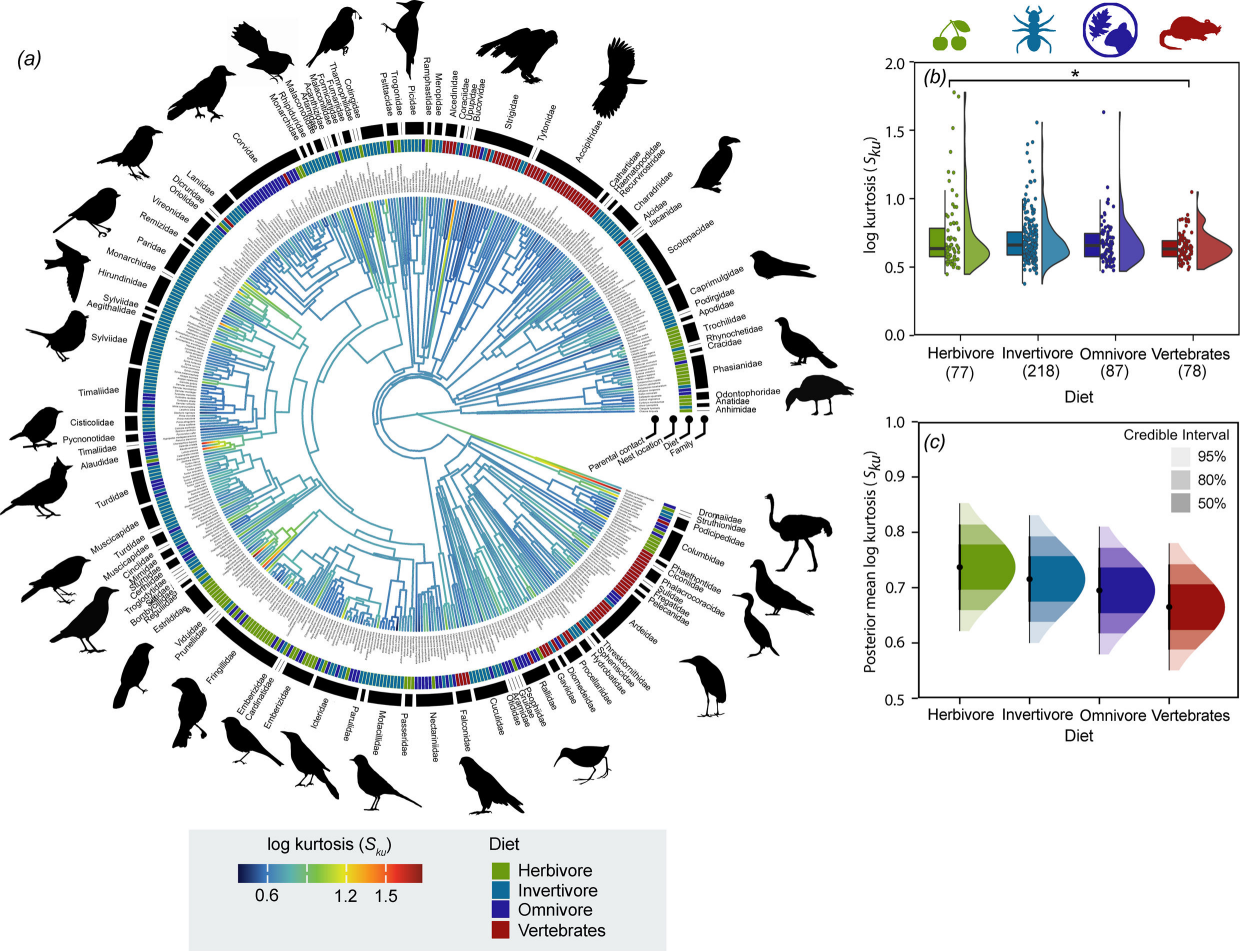
Phylogenetic tree and significant predictors of eggshell surface kurtosis (*S_ku_*) across 460 bird species. (*a*) Branch colours show the diversification in *S_ku_* across the phylogeny and branch lengths show ancestral trait estimates. In the hybrid box plot (*b*), *S_ku_* of eggshell surfaces was higher in species with a plant-based diet compared with primarily vertebrate-based diet. *S_sk_* are shown as filled circles, vertical line indicates the median, box shows the interquartile range (IQR) and the whiskers are 1.5 × IQR, and their distribution are shown as histograms. Significant differences between categorical variables based on MCMCglmm are given by asterisks with *p < 0.05. (*c*) Density plot of posterior predictions from species diet, the only significant predictor in the MCMCglmm model. The plot shows median posterior values with 50%, 80% and 95% credible intervals, illustrating uncertainty around predicted log *S_sk_* values for each diet category. Silhouette illustrations came from PhyloPic (http://phylopic.org), contributed by various authors under public domain licence (see electronic supplementary material).

Kurtosis is the measure of the sharpness of the peaks of the surface, with the sharpest peaks being found in common ostriches (*S_ku_* = 55.65, *n* = 1), Eurasian hoopoes (*S_ku_* = 24.95 ± 10.48, *n* = 5) and black-necked grebes (*S_ku_* = 15.82 ± 11.03, *n* = 2). Only five species had *S_ku_* < 3, meaning that their eggshells are not characterized by sharp peaks or troughs. Peakedness of eggshell surfaces across the avian species were higher for species with plant-based diets compared with those with primarily vertebrate-based diets (MCMCglmm: *p* = 0.02, figure 5*a,b*, electronic supplementary material, table S11). The distribution of posterior means revealed a gradual decrease in *S_ku_* values across diet categories, progressing from herbivores to invertivores, omnivores and finally vertebrate-based diets (figure 5*c*).

## 4. Discussion

Overall, the phylogenetic signal for the three measured traits—surface roughness (*S_a_*), surface skewness (*S_sk_*) and surface kurtosis (*S_ku_*) [60]—was intermediate to low, as indicated by Pagel’s *λ* values for all three traits significantly differing from 1 but not 0. A *λ* of 1 would indicate a Brownian motion model of trait evolution, where the phylogeny accurately reflects the covariances between species for a given trait [61,62]. This finding suggests that life-history traits, therefore, are largely more influential determinants of eggshell roughness characteristics than relatedness. Similar observations regarding phylogeny and eggshell parameters have been reported for eggshell wettability [53], gas conductance [13] and background and foreground texture comparisons [14], in contrast to eggshell calcium content [63] and eggshell thickness [64], where the phylogenetic signal was high.

### 4.1. Surface roughness (*S_a_*)

Across all bird species measured, eggshell maculation and nest type (see table 1) were the strongest determinants of eggshell surface roughness (*S_a_*). Species with maculated eggshells had significantly higher *S_a_* than species that lay immaculate eggs; immaculate eggs generally have a smoother eggshell surface. Smoother surfaces have less surface area compared with rough surfaces, which can reduce the number of sites available for bacterial adhesion, and are easier to keep clean [60]. Smoother surfaces also promote the flow of water, preventing the formation of biofilms [65]. Many immaculate eggs, particularly those which are primarily white, are typically found in cavity-nesting species, where humidity and risk of bacterial infection are high [66]. Smoother surfaces with good wettability may assist in minimizing potential bacterial infections. However, smooth surfaces may still support the formation of biofilms if they are regularly covered in detritus and/or faeces [67]. A different subset of bacteria is thought to proliferate in cavity-nesting environments compared with more exposed nests [66], which may lead to the evolution of different eggshell anti-microbial defence mechanisms targeted towards specific microbiota. For example, the avian eggshell pigment protoporphyrin (present in very low concentrations in white eggshells) has a light-activated antimicrobial function against gram‐positive bacteria [66], which would benefit eggs frequently exposed to sunlight. The adhesion properties of the two types of bacteria (gram-positive and gram-negative [68]) often differ [69], and their ability to adhere to different eggshell surface roughness warrants further investigation.

### 4.2. Surface skewness (*S_sk_*)

We found that approximately 91% of the species analysed produce eggshell surfaces with negative *S_sk_*, suggesting these surfaces are mainly composed of valleys rather than peaks (see figure 1). Various engineering experiments have demonstrated that negatively skewed surfaces may perform better in abrasive environments [70] because the valleys can trap wear particles (debris, or particles, of various size, shape, colour distributions and chemical compositions). Most nests, regardless of their location, will have some degree of abrasiveness. While this may be mediated by nest material and lining, there will still be contact between the eggs and a substrate.

Higher *S_sk_*—not necessarily negative or positive—can have numerous benefits. Such benefits observed in engineering include better wear resistance [71], improved contact area [46], improved heat dissipation [60], increased tangential stiffness [72] and improvements in elastic deformation [73]. Higher *S_sk_* is associated with better wear characteristics, permitting any peaks to wear down initially, leaving a more stable surface within the valleys. Such a mechanism may offer benefits to eggs, as the outer edge of the surface wears down during the incubation process due to friction between the egg and the incubating parent, other eggs and nest substrate [74]. Generally, when two bodies with curved surfaces, such as eggs, are brought into normal contact and then displaced in a tangential direction relative to one another, they tend to stay adhered in parts of the contact area while slipping in other areas relative to each other [75]. A negative skewness typically improves the contact of rough surfaces; it also leads to friction reduction [46] and decreased wear of smooth and rough surfaces under dry sliding conditions (i.e. when two surfaces slide over each other) [76,77]. For the small percentage of species (*ca* 7%) that have a positive *S_sk_*, an additional benefit is a reduced contact area between the eggshell and the nest and/or substrate, which can reduce wear and friction [39]. Lastly, surfaces with greater positive *S_sk_* beyond a certain roughness can have more effective heat dissipation [78]. These improved heat dissipation properties are due to the increased surface area provided by the peaks and valleys, and could potentially be particularly advantageous for species nesting in exposed locations.

### 4.3. Surface skewness (*S_ku_*)

None of the variables we investigated were significant determinants of the *S_ku_* of eggshell surface structure for the species we measured. Given that Pagel’s *λ* is low for *S_ku_* (*λ* = 0.21; S*_sk_* is lower), this suggests that undocumented life-history traits probably influence *S_ku_*, as relatedness does not drive this facet. Potential undetermined life-history traits could include more detailed aspects of parental incubatory behaviour, subtle differences in nesting material substrates as opposed to broad nest types, and the ratio of eggshell maculated foreground to the background base colour. Rough surfaces with high *S_ku_* have higher and sharper peaks, contributing to an increase in contact pressure and a decrease in contact area [39]. However, the impact of *S_ku_* becomes minimal at *S_ku_* ≥ 5 as there is less variation in contact area and pressure, and overall, less friction [79,80]. In our study, 33% of species had mean eggshell *S_ku_* ≥ 5, suggesting that this variable may not be functionally important for at least some species.

### 4.4. Future directions

At the time this study was conducted, the phylogeny from BirdTree represented the most complete molecular phylogeny of extant birds [81]. Since then, a new consensus bird tree has emerged, which incorporates phylogenetic estimates for 9239 species from 262 studies published between 1990 and 2024, with additional species positioned based on curated taxonomic information [82]. This updated consensus tree may offer fresh insights into avian phylogeny and could potentially reshape the interpretation of our findings. Future research could benefit from utilizing this latest phylogenetic framework to explore how these advancements impact our understanding of eggshell surface texture across different bird species.

All the eggs measured in the present study were probably freshly laid, based on the small size of their blow holes, and the tendency for historical egg collectors to collect clean freshly laid eggs [83]. Previously, it has been demonstrated that as the incubation period advances, the height distribution of peaks and depressions on the inner surface of chicken (*Gallus gallus*) eggshells shifts from a roughly symmetrical pattern to one that is skewed above the mean plane by the time of hatching. Our study focused on the exterior surface of eggshells, which are known to erode over the course of the incubation process [41,74]. A fruitful avenue of further investigation would be to track the changes in *S_a_*, *S_sk_* and *S_ku_* throughout incubation, to determine how these parameters change and interact over the incubation process.

## Data Availability

The data and code used for statistical analysis are available at: https://figshare.com/s/1dbaa5ae26554677473d. Statistical outputs are provided in the electronic supplementary material. The data are provided in electronic supplementary material [78]. Supplementary material is available online [84].
